# Prevalence of Depression and Associated Factors among Adults in Saudi Arabia: Systematic Review and Meta-Analysis (2000-2022)

**DOI:** 10.1155/2023/8854120

**Published:** 2023-09-14

**Authors:** Mohamed O. Nour, Khulud K. Alharbi, Tamara Abdulrahman Hafiz, Ali M. Alshehri, Lujain Sami Alyamani, Taif Hazzaa Alharbi, Reuof Saleh Alzahrani, Ebtehal Fawwaz Almalki, Asayel Atiah Althagafi, Ebtesam Tariq Kattan, Hala Mamun Tamim

**Affiliations:** ^1^Department of Health Promotion and Education, Faculty of Public Health & Health Informatics, Umm Al-Qura University, Makkah, Saudi Arabia; ^2^Department of Public Health and Community Medicine, Damietta Faculty of Medicine, Al-Azhar University, Egypt; ^3^Department of Health Services Management, Faculty of Public Health & Health Informatics, Umm Al-Qura University, Makkah, Saudi Arabia; ^4^Department of Environmental Health, Faculty of Public Health & Health Informatics, Umm Al-Qura University, Makkah, Saudi Arabia

## Abstract

**Introduction:**

Depressive disorders are the leading causes of disability and disease burden worldwide. They were ranked fifth among the top causes of death and disability in Saudi Arabia, with subsequent impacts on productivity and economics when affecting adults.

**Objectives:**

The study aimed to systematically investigate the prevalence of depression and its associated factors among Saudi adults during 2000-2022.

**Methods:**

A systematic review and meta-analysis were conducted in accordance with the PRISMA guidelines using Medline/PubMed, ResearchGate, Google Scholar, and Ovid databases during 2000-2022 with predetermined inclusion and exclusion criteria. The quality of the included papers was determined, and heterogeneity between studies was assessed using the *Q* statistic to estimate the *I*^2^ value. A random effects model was used to drive the pooled depression prevalence with 95% confidence intervals (CI). A forest plot was generated to show estimates for individual studies. Publication bias was assessed visually with the funnel plots symmetry and Egger's test (*p* < 0.05). A sensitivity analysis was conducted to explore the effect of individual studies on the overall prevalence estimate. A subgroup analysis by the study, population characteristics, and depression tools were also run.

**Results:**

Forty-six cross-sectional relevant studies were identified, including 25814 participants. The pooled depression prevalence was 37.35% (95% CI: 33.61–41.98%) with high heterogeneity (*I*^2^ = 94.8%, *p* < 0.001). In the subgroup analysis, the prevalence estimates were higher among females (34.5%), the singles (49.5%), undergraduate university students in university settings (47.7%), northern region (62.3%), and studies that utilized the Center for Epidemiological Studies-Depression (CES-D) scale (58.8%). Among the risk factors significantly associated with depression were female gender, being single, low education level, financial problems, poor housing condition, having medical problems, sleep disorders, presence of psychiatric/psychological conditions, life events, lack of social support, exposure to stress, educational/personal problems, and smartphone addiction.

**Conclusion:**

Almost more than one-third of Saudi adults had depression. Appropriate surveillance, early interventions, and depression management strategies are needed to decrease the prevalence and its consequences among adults.

## 1. Background

Depressive disorders are the leading causes of disability and disease burden worldwide and are major underlying causes of self-harm. They affect all types of people—young and old, rich and poor—in all countries. Globally, it is estimated that 5% of adults suffer from depression [1]. According to the Global Burden of Diseases Study (GBD) in 2019, the global prevalence of depressive disorders has been increasing in recent decades from 170.8 (152.7–190.4) million in 1990 to 279.6 (251.6–310.3) million in 2019. They ranked sixth among the top worldwide causes of disability-adjusted life-years (DALYs) in the 25–49-year age group and the largest proportion of mental disorder DALYs (37.3% (32.3–43.0)). They are considered the most common mental disorder across both sexes and years; however, their burden was greater in females than males. In North Africa and the Middle East, the prevalence of depressive disorders was high, accounted for 4348.9 cases (3807.3–4971.1) per 100 000 people [2, 3].

In Saudi Arabia in 2019, they ranked fifth among the top causes of death and disability (DALYs) in all ages combined. The age-standardized prevalence per 100 000 was 3148.3 (2664.7–3700.6), and the age-standardized DALYs per 100 000 was 1846.3 (1356.1–2433.4) [3].

According to the preliminary population estimates in the mid-2021, the proportion of Saudi adults aged 18-60 years was approximately two-thirds of the total Saudi population [4]. This age group is characterized by achieving autonomy, establishing identity, and developing emotional stability and is considered the backbone of economic development [5]. If they are exposed to psychiatric problems such as depressive symptoms, their physical and cognitive health will be impaired with subsequent impact on daily life function and economic loss. In addition, depression is closely related to the development of chronic physical illnesses such as diabetes, cardiovascular diseases, asthma, and arthritis, as well as having a poor prognosis [6].

There is a great variability in studies reporting the prevalence of depression among adults in Saudi Arabia. Therefore, understanding and estimating its prevalence and identifying the associated risk factors are necessary for early detection, improve care, and guide future research in this population. In addition, systematic evidence collection is important and can refine such evidence in order to facilitate depressive disorder prevention and promotion programs among this cohort. Therefore, this systematic review and meta-analysis investigates the magnitude of depressive disorders and their associated factors among adult general population in Saudi Arabia during 2000-2022.

## 2. Methods

### 2.1. Study Design

A retrospective database analysis, systematic review, and meta-analysis of related studies were utilized. It was part of a wider project targeting different epidemiological and public health aspects relevant to depressive disorders in Saudi Arabia since the year 2000.

### 2.2. Research Questions

Targeting depression as a “condition”, Saudi Arabia as a “context”, and depression among Saudi adults (18-60 years), both genders, and nonrisky groups as a “population”, the research questions of that review were (a) “What is the prevalence of depression among Saudi adults (18-60 years) during 2000-2022?” and (b) “What are the associated risk actors of depression among this group during 2000-2022?”

### 2.3. Types of Included Studies

Different observational study designs were used (cross-sectional, epidemiological surveys, prospective and retrospective cohorts, surveillance studies, etc.), as well as epidemiological data generated by the WHO country profile and international and governmental records that reported the prevalence and associated risk factors of depression among adult Saudi population (18-60 years).

### 2.4. Search Strategy

We followed the Preferred Reporting Items for Systematic Reviews and Meta-Analyses (PRISMA) guidelines [7], and the study protocol was prospectively registered with PROSPERO (CRD42022374628) and approved by the Bioethics Committee at Umm Al-Qura University, Saudi Arabia (HAPO-02-K-012-2022-03-1000).

We consulted an expert librarian to improve and sharpen the search strategy for studies published from 1^st^ January 2000 to 31^st^ December 2022. We searched four selected bibliographic electronic databases including Medline/PubMed, ResearchGate, Google Scholar, and Ovid. Other sources included the Saudi Digital Library (SDL) for unpublished studies and dissertations and online relevant Saudi journals (as Saudi Medical Journal, Annals of Saudi Medicine, Journal of Family and Community Medicine, Journal of Infection and Public Health, Saudi Journal of Medicine and Medical Sciences, etc.). We tried to consult experts in psychiatry and neurology care at the Saudi Ministry of Health (MOH) and local universities for additional research papers. The reference list of selected papers was further hand-searched for additional relevant eligible studies. We tried, through official emails and personal communications, to contact local authors of dissertations for clarification when outcome data are missing, or the methodology was unclear.

The search strategy included the following keywords in different combinations (using “OR” then “AND”) depending on the applied database: “Prevalence”, “Epidemiology”, “Depression” or “Depressive symptoms” or “Mental disorders”, “Adult” or “General population” or “Public”. This string was further attached to Saudi Arabia and the years from 2000 to 2022, and all search terms were searched in both title, abstract, and keywords. All systematic reviews regarding depression in Saudi Arabia were reviewed for eligible studies.

### 2.5. Main Outcomes

Prevalence and associated risk factors related to depression among adults in Saudi Arabia. We utilized the WHO standard definition for depression [1] based on different valid measurement tools.

### 2.6. Inclusion Criteria

Full-text published online studies and reports, unpublished studies and dissertations from the SDL, and articles published in English language, with a sample size of at least 100 participants to ensure an adequate level of statistical power and robustness in our analysis and to avoid selection bias from small studies, targeting prevalence and associated risk factors of depression in Saudi Arabia, on the Saudi adult general population, aged 18-60 years, and during the specified period from 1^st^ January 2000 to 31^st^ December 2022.

### 2.7. Exclusion Criteria

We excluded citations without full text, case reports or case series, preprints, protocols, commentaries, letters to editor, conference proceedings or abstracts, systematic reviews and meta-analyses, studies conducted outside Saudi Arabia or conducted on a non-Saudi population, studies targeting risk groups as pregnant and hospitalized patients and those with known mental or psychological disorders, studies investigating COVID-19-related depression, studies without primary data or with duplicate data, or without a sampling method.

### 2.8. Data Extraction (Selection and Coding)

We used EndNote X9 to remove duplicates. Then two independent reviewers (MA and BQ), blinded to each other's decisions, manually screened the titles and abstracts of the articles for inclusion. The final selected articles were divided into two halves, with each half being screened and read for the full text by one independent reviewer and a member of the research team. A standardized eligibility form containing the inclusion and exclusion criteria was tested (as a pilot) on 10 studies and modified accordingly. This form was used by each team to record their decisions and comments for each article and the reasons for exclusion. Article coding was classified as “included,” “excluded,” or “not sure.” “Excluded” articles by both teams were eliminated from the review. Discrepancies were resolved by consensus or by a third reviewer (TS).

If a paper described outcomes in multiple regions within the country, the data from each region were reported separately if available. If supplementary files for included articles were available, they were reviewed for relevant information. Data from selected articles were manually recorded using an Excel spreadsheet.

The data extracted from each study included: the name of first author; year of publication; period of data collection; study location (region); study design; study setting; target population; response rate; sample size (total population screened); depression tool used; prevalence (number of depression cases graded from mild to severe/extremely severe according to the measurement tool or depression scale used); and population characteristics (both total and those with depression) like age, gender, and marital status.

**Figure 1:**
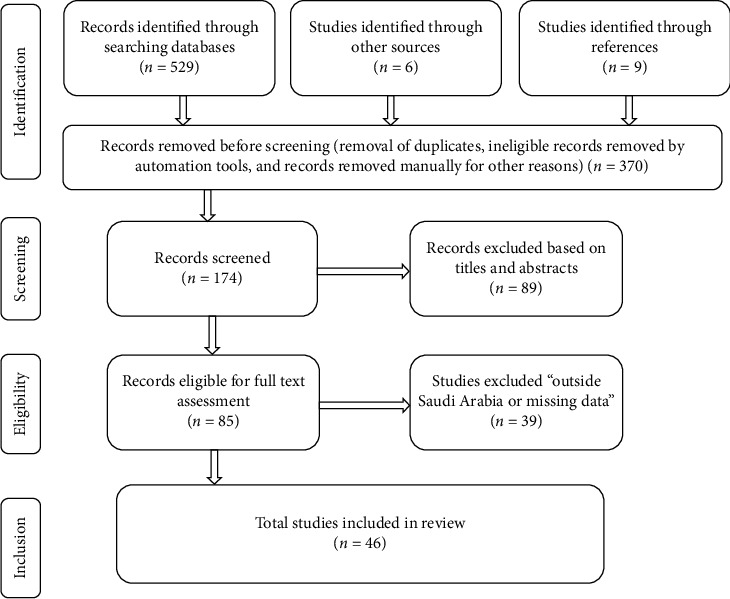
The PRISMA flow chart for study search and selection process.

A PRISMA flow chart was done to document the number of studies included and excluded at each stage of the study selection process (Figure 1).

### 2.9. Quality (Risk of Bias) Assessment

Guided by methods used in previous systematic reviews, we used a modified version of the Newcastle-Ottawa scale [8] to evaluate the risk of bias and the quality of the included papers. It depends on adequate participant selection (0–4 points), comparability of studies based on design and analysis (0–1 point), and adequate ascertainment of outcomes (0–3 points). So, the total score ranged from 0 to 8. Studies with a score “6–8” were considered good quality, those with a score “3–5” as moderate quality, and those with a score “0–2” as low quality. We excluded low-quality studies (high risk of bias) as they can introduce bias and confound the findings and to ensure that the included studies were of sufficient quality to provide reliable and valid results. We identified 13 studies with high quality (low risk of bias) and 33 studies with moderate quality (moderate risk of bias). The scores of the papers are presented in Table 1.

### 2.10. Strategy for Data Synthesis

Tables and figures were used to illustrate summary results including key study and participant characteristics with descriptive statistics of frequencies and percentages. Heterogeneity and variability in results among the selected studies should be suspected as studies may differ in design, sampling, methodology, and individual characteristics. So, studies were assessed using the *χ*^2^ test on Cochran's (*Q*) statistic to estimate the *I*^2^ value, which refers to the percent variation across studies that is due to heterogeneity (true between-study differences), rather than sampling error or chance [9]. Heterogeneity was considered significant with *p* < 0.1, and it was categorized as low, moderate, and high when the *I*^2^ values were <25%, 25%–75%, and >75%, respectively. A random-effects meta-analysis model [10] with the DerSimonian–Laird method was used to determine the pooled measures (combined data from all included studies to estimate the pooled prevalence of depression) with 95% confidence intervals (CI). The prevalence was adjusted in a multivariate meta-analysis considering the variance between different tools used to measure depression. A forest plot was generated to show estimates for individual studies. Publication bias, the tendency to publish studies with beneficial outcomes or studies that show statistically significant findings, was assessed visually with the funnel plot symmetry [11], and Egger's test (*p* < 0.05) was also indicative of publication bias for small study effects. A sensitivity analysis was conducted to explore the effect of individual studies on the overall prevalence estimate by serially excluding each study. We considered subgroup analyses by the study, population characteristics, and depression tools.

## 3. Results

### 3.1. Study Selection

We identified 544 studies (529 from electronic database searches, 9 from references, and 6 from other sources). After removing duplicates, those published before the year 2000, and ineligible records (*n* = 370), we screened the titles and abstracts of 174 studies of which we excluded 89 studies. We retrieved the full texts of the remaining 85 studies and excluded 39 of these studies because they did not report on the prevalence of depression, missing essential data, or not done in Saudi Arabia. We included a final total of 46 studies [12–57] that met eligibility requirements. They were of moderate (*n* = 33) or high quality (*n* = 13). The study search and selection process are shown by the PRISMA flow chart in Figure 1. The prevalence of depression was reported by all studies and the degree of depression by 37 studies. The extracted data from the selected studies are summarized in Table 1.

**Figure 2:**
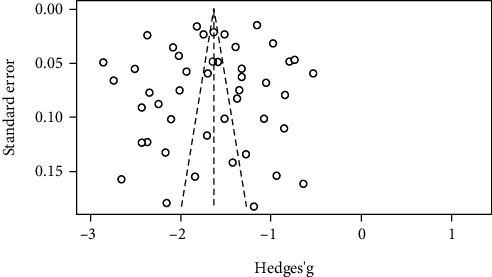
Funnel plot of studies reporting the prevalence of depression.

The funnel plot was relatively symmetric, and the results of Eggers' test (*p* = 0.537) and Begg's rank test (*p* = 0.462) were nonsignificant indicating the absence of publication bias for the studies reporting the prevalence. (Figure 2) Sensitivity analysis by serial repetition of the meta-analysis after excluding each study suggested that no individual study affected the overall prevalence estimate by more than 1%.

### 3.2. Characteristics of Included Studies

All studies utilized a cross-sectional study design, published between 2007 and 2022, and the data included were collected between 2007 and 2021. Three studies [12, 42, 44] were nationwide (*n* = 3359) and the remaining 43 were reported from different regions within Saudi Arabia (northern (3 studies, *n* = 716), southern (5 studies, *n* = 1786), eastern (3 studies, *n* = 7395), western (14 studies, n =5523), and central (18 studies, *n* = 7035) regions). Nine (out of total 13) administrative areas were represented in the studies (Riyadh (15 studies, *n* = 6309), Makkah (14 studies, *n* = 5523), Eastern (3 studies, *n* = 7395), Qassim (3 studies, n =726), Jazan (2 studies, *n* = 1114), Tabuk (2 studies, *n* = 561), Albaha (2 studies, *n* = 400), Hail (one study, *n* = 155), and Najran (one study, *n* = 272)). However, the Northern Borders, Madinah, Al-Jawf, and Asir areas were not represented by separate studies. About one third of the studies were conducted in the capital city, Riyadh (15 studies, *n* = 6309), followed by Jeddah (7 studies, *n* = 2256), and Makkah city (4 studies, *n* = 1232).

The study data were collected in various settings including universities (25 studies, *n* = 12611), healthcare (11 studies, *n* = 3975), community/public settings (9 studies, *n* = 8871), and one study (*n* = 357) was conducted at King Fahad Air Base that was later considered as public setting in subgroup analysis. The actual survey method was online in 28 studies.

Different screening tools were used to measure depression including the Beck Depression Inventory (BDI) (17 studies, *n* = 7979), the Patient Health Questionnaire (PHQ-9) (17 studies, *n* = 13403), the Depression Anxiety and Stress Scale (DASS-21) (7 studies, n =2612), the Hospital Anxiety and Depression Scale (HADS) (3 studies, n =1399), the Aga Khan University Anxiety and Depression Scale (AKUADS) (one study, *n* = 302), and the Center for Epidemiological Studies-Depression (CES-D) (one study, *n* = 119).

There are also disparities in the assessment criteria for prevalence; that is, 17 studies (*n* = 6705) considered (“mild,” “moderate,” “moderately severe” to “severe/extremely severe”) for identifying prevalence rates, whereas 18 studies (*n* = 10949) considered (“mild”, “moderate” to “severe/extremely severe”), one study (*n* = 305) considered (“moderate”, “moderately severe” to “severe/extremely severe”), one study (*n* = 119) considered (“mild/moderate” to “severe/extremely severe”), and nine studies (*n* = 7736) did not clearly report the cutoff points.

The response rate of the included studies ranged from 13% to 100%. In 7 studies, the total number of participants exceeded the predetermined sample size (100%+) due to the online nature of data collection, a convenient sampling method, or purposively to compensate for any missing data.

Of the 46 studies, the total study population was 25814 of which 13918 (53.9%) were females and 11896 (46.1%) were males. They ranged from 119 participants in a study conducted in Qassim, Central Region [13] to 5172 participants in a study conducted in Al-Ahsa, Eastern Region [37]. The mean age of participants was reported in 32 studies that ranged from 20.58 ± 1.71 to 47.5 ± 13.9 years, whereas 14 studies did not report the mean age of their subjects. Four studies (*n* = 1168) were conducted on female participants only, three studies (*n* = 912) on male participants only, and the remaining 39 studies (*n* = 23734) included both genders. They included undergraduate university students in 25 studies (*n* = 12611; 48.9%), general population in 9 studies (*n* = 8871; 34.3%), healthcare workers (HCWs) in 11 studies (n =3975; 15.4%), and military personals in one study (*n* = 357; 1.4%).

Regarding participants with depression (n =9635), 28 studies (*n* = 6358) provided data on their gender (2869 males; 45.1% and 3489 females; 54.9%), 9 studies (*n* = 912) on age (18-60 y), and 14 studies (*n* = 1850) on marital status (1280 single; 69.2%, 526 married; 28.4%, and 44 others; 2.4%).

### 3.3. Prevalence of Depression

**Figure 3:**
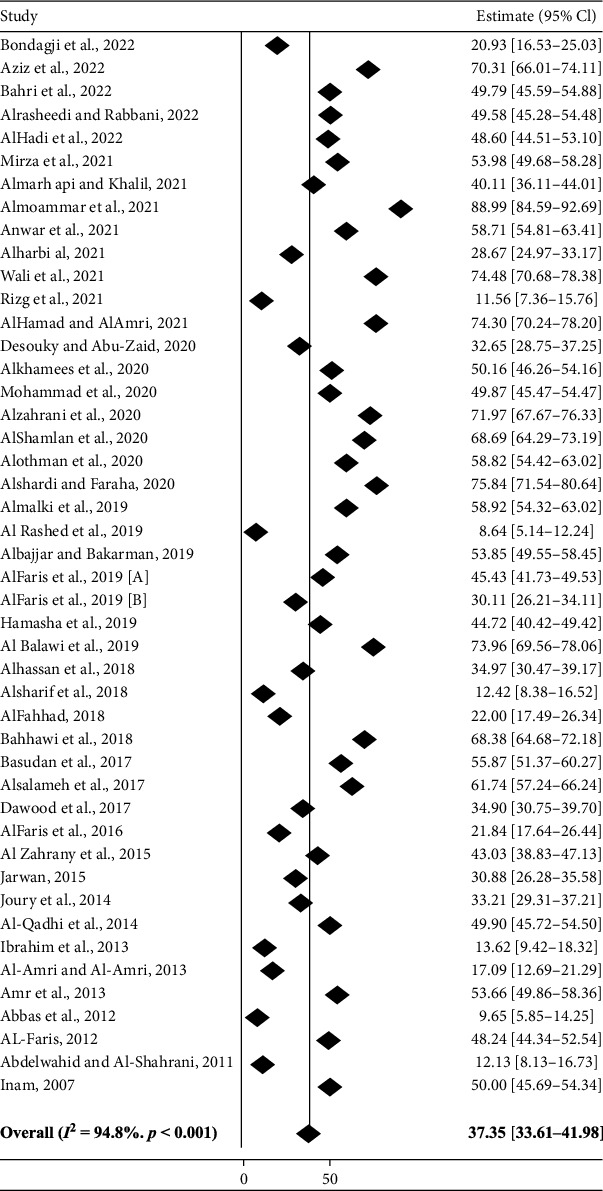
Forest plot for the prevalence of depression among Saudi adults.

The overall prevalence estimates of depression among Saudi adults reported by the 46 studies yielded a pooled prevalence of 37.35% (95% CI: 33.61–41.98%). The adjusted prevalence considering the variance between different measurement tools was 35.84% (95% CI: 30.28–42.15%) with 46.3% of variance due to difference between measurement tools. The prevalence ranged from 8.64% (95% CI: 5.14–12.24%) in a study in Al-Ahsa [37] to 88.99% (95% CI: 84.59–92.69%) in a study in Albaha [22], with high heterogeneity (*I*^2^ = 94.8%, *p* < 0.001), and the variance between the studies was slightly high (*τ*^2^ = 0.38). (Figure 3).

### 3.4. Subgroup Analysis of Studied Variables

There were no sufficient data to pool the prevalence of depression among different age groups; however, subgroup analyses by gender, marital status, target population, study setting, study region/area, and screening tool were conducted to explore potential heterogeneity between studies.

Regarding gender, the total study population was 25814 of which 13918 (53.9%) were females and 11896 (46.1%) were males. Four studies (*n* = 1168) were conducted on female participants only, three studies (*n* = 912) on male participants only, and the remaining 39 studies (*n* = 23734) included both genders. Twenty-eight studies (*n* = 6358) provided data on the gender of participants with depression where 2869 were males (45.1%) and 3489 were females (54.9%). The prevalence estimates of depression were slightly higher among females (34.5%, 95% CI: 30.1–39.8%, *I*^2^ = 97.1%) than males (32.7%, 95% CI: 27.9–37.2%, *I*^2^ = 96.3%).

Regarding marital status, fourteen studies (*n* = 4822) reported data on the marital status of participants with 2139 (44.3%) were married, 2588 (53.7%) were singles, and the remaining 95 (2%) were others. The prevalence of depression was reported among 1850 participants of whom 1280 were singles (69.2%), 526 were married (28.4%), and 44 were others (2.4%). The prevalence estimates of depression were higher among the singles (49.5%, 95% CI: 45.6–55.2%, *I*^2^ = 90.3%) than the married (24.6%, 95% CI: 19.8–30.1%, *I*^2^ = 88.7%).

Regarding the study settings and the target population, 25 studies (*n* = 12611) were conducted in universities targeted undergraduate university students, 11 studies (*n* = 3975) were conducted in healthcare settings targeted HCWs, and 10 studies (*n* = 9228) were conducted in community/public settings targeted the general population. The prevalence of depression was reported among 9635 participants of whom 6015 (62.4%) were undergraduate university students in university settings, 2218 (23.0%) were general population in community/public settings, and 1402 (14.6%) were HCWs in healthcare settings. The prevalence estimates were higher at studies conducted among undergraduate university students in university settings (47.7%, 95% CI: 41.0–54.5%, *I*^2^ = 95.1%), followed by HCWs in healthcare settings (35.3%, 95% CI: 30.9–41.4%, *I*^2^ = 92.6%), whereas the least prevalence was in studies conducted among the general population in community/public settings (24.0%, 95% CI: 19.8–30.1%, *I*^2^ = 93.6%).

For the study region/area, 3 studies were nation-wide (*n* = 3359), and the remaining 43 studies were reported from different regions within Saudi Arabia: 3 studies (*n* = 716) in the northern region, 5 studies (*n* = 1786) in the southern region, 3 studies (*n* = 7395) in the eastern region, 14 studies (*n* = 5523) in the western region, and 18 studies (*n* = 7035) in the central region. The prevalence estimates were higher at northern region (62.3%, 95% CI: 58.2–67.0%, *I*^2^ = 96.6%) followed by southern region (55.9%, 95% CI: 51.8–60.7%, *I*^2^ = 95.5%), whereas the least depression prevalence was at eastern region (23.2%, 95% CI: 19.7–27.1%, *I*^2^ = 89.6%).

**Figure 4:**
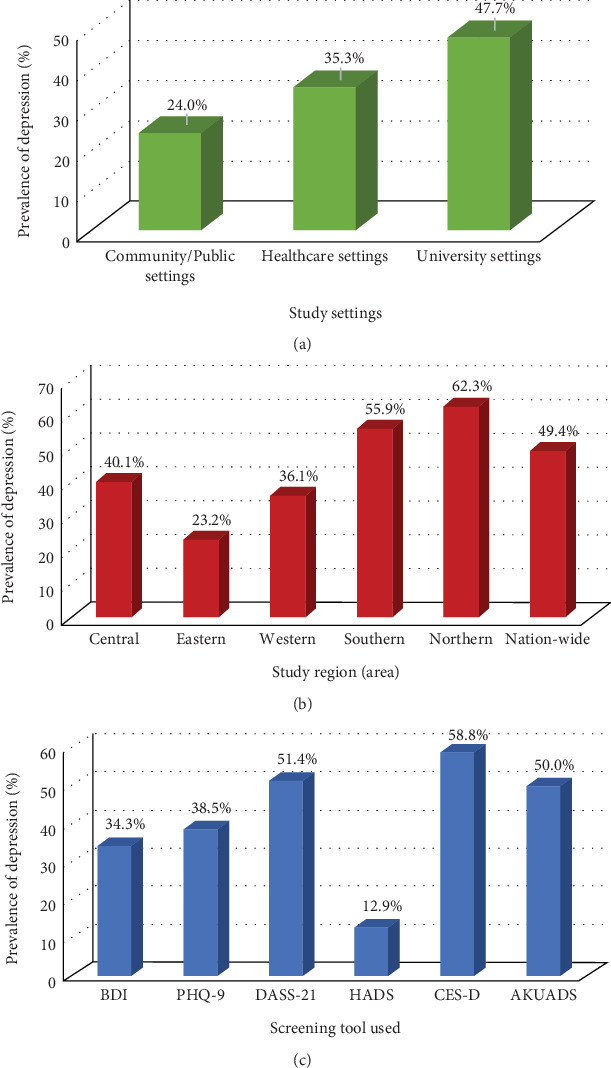
Bar graph of meta-analysis of the prevalence of depression among Saudi adults stratified by (a) study settings, (b) study region/area, and (c) screening tool used.

Regarding the screening tools that were used to measure depression, the BDI was used in 17 studies (*n* = 7979), the PHQ-9 in 17 studies (*n* = 13403), the DASS-21 in 7 studies (*n* = 2612), the HADS in 3 studies (*n* = 1399), the AKUADS in one study (*n* = 302), and the CES-D in one study (*n* = 119). The highest depression prevalence estimates were found among studies that used CES-D (58.8%, 95% CI: 53.5–63.2%, *I*^2^ = 93.3%) followed by DASS-21 (51.4%, 95% CI: 46.8–55.1%, *I*^2^ = 96.0%), whereas the least depression prevalence was among studies that used HADS (12.9%, 95% CI: 9.4–16.6%, *I*^2^ = 95.1%) (Figure 4).

### 3.5. Associated Factors and Predictors of Depression among Saudi Adults

Of the 46 studies included in this meta-analysis, only 24 reported on significant factors associated with or predictors of depression among Saudi adults as illustrated in Table 2. Sociodemographic factors associated with depression included female gender [12, 14, 15, 17, 24, 25, 27, 30, 51, 57], being single [25], having only female siblings [52], low education level [37, 42, 43], occupational status (employee vs. nonemployee) [56], financial problems [32, 51], and poor housing condition [56].

Health-related factors found to be related to depression included having medical problems [25], sleep disorders [16, 32], and presence of psychiatric/psychological conditions [24, 33].

Life events and social life have been related with depression, including the loss of a first-degree relative or beloved person in the last year [23, 33, 47, 53], loneliness [16], disturbed marriage [32], presence of family conflicts [24], emotional failure or exhaustion [30, 53], poor social life or lack of social support [32, 33, 43, 53], having problems with relationships with others [45, 52], and exposure to high cynicism [30].

Other factors included exposure to stress (as work stressors) [15, 16, 25, 28, 32, 37], presence of anxiety [53], educational problems [13, 24, 27, 43, 51, 53], personal problems [51], and history of depression [32, 47]. The association between depression and both social media usage [17] and smartphone addiction [42] was also reported.

## 4. Discussion

This systematic review explored and summarized the literature related to depression among Saudi adults over the past two decades. Our review included 46 studies with 25814 participants.

### 4.1. Prevalence of Depression

Our data revealed a pooled prevalence of depression of 37.35% ranged from 8.64% to 88.99%. This finding was higher than the prevalence in nearby Gulf countries: 4.2%-6.6% among general population in Qatar [58], 4.0% and 7.4% as 12-month and lifetime prevalence among the Iraqi general population [59], 12.5%-28.6% among both citizens and expatriates in the United Arab Emirates [60], and 27.7% among university students in Oman [61].

Given the data available from our analysis, it is undeniable that variability in depression prevalence is noticed in different regions within the country. The northern and southern regions in particular are high-burden regions where depression is prevalent at a discouragingly higher rate than other regions. This may be attributed to a combination of reasons like socioeconomic, demographic, and ecological circumstances. Of further interest, we found that undergraduate university students and studies conducted in university settings showed the highest prevalence estimates. The subsequent effects on this group of population are worrying, and therefore, it is critical to develop plans for early detection and management of depression in academic environments. Similar findings were reported in the literature that linked depression to academic and study difficulties [62–64].

There is large heterogeneity in study measurement with no standard approach for identifying cut-off points for each of the depression tools used in these studies, which may have resulted in the wide range estimate across the studies, even among those using the same instrument, that makes the comparison of findings between and across studies difficult, and accordingly, the results should be interpreted with caution considering the potential impact on the validity of the findings.

### 4.2. Risk Factors and Predictors of Depression

Aletesh et al. assessed the degree of public perception and attitudes toward depressive disorders in Saudi Arabia. Positive comments were received from the general public regarding awareness of depression and its manifestations. However, the awareness of risk factors and managements was poor [65].

Consistently, higher estimates of depression were reported for females than males in most of the studies. This agrees with what has previously been observed in a systematic analysis for the GBD 2019 [3] and in a systematic review in 2020 among Saudi medical students, in which they reported that females were at a higher risk of depression [66]. The differences in socioeconomic factors, such as education, income, culture, diet, and abuse, may affect the higher depression rate in female. However, biological sex differences and changes in ovarian hormones could contribute to the increased prevalence in female [67].

There is evidence that the prevalence of depression tends to rise with aging in women [68]. The increased likelihood of elderly people developing or progressing in comorbidities, losing a spouse, losing dear friends or family members, or experiencing disintegration of the family composition with the detaching of sons and/or daughters for marriage or travel are just a few of the factors Kurtz et al. attributed to this phenomenon [69].

Another sociodemographic factor associated with depression was being single when compared to being married since living alone is a predisposing factor. Beutel et al. found that loneliness poses significant health risk for depression (OR = 1.91, 95% CI: 1.74-2.09, *p* < 0.001) [70]. Depression was also associated with level of education, occupational status, financial problems, and poor housing conditions. In this regard, according to Bauldy's theory, persons who come from impoverished origins may be better protected from depression by having a good education than those who come from advantaged homes [71]. That was later explained by Ross and Mirowsky, who stated that “people from disadvantaged backgrounds are more likely to lack a sense of mastery and self-efficacy” [72]. In the same context, Bauldry assumed that the job might act as a moderator in the relationship between high education level and depression; for instance, the likelihood of depression increases in people with a high level of education when they are given jobs that are beneath their qualifications or in those who are unemployed. [71]. According to the findings, people without job were more likely than those with jobs to have depression symptoms.

Similar to our findings, Mohamed et al. found that people who had stressful experiences, those who reported sleep difficulties, and those with a positive family history of depression were much more likely to suffer depression than those who had not [73]. Our analysis showed financial troubles as a risk factor for depression. In their systematic review, Guan et al. found that financial stress is positively associated with depression among adults in both high-income and low-and middle-income countries that can be explained by social causation, psychological stress, and social selection pathways [74]. Poor housing conditions were also reported as predictors for depression. In their systematic review, Singh et al. found a positive association between at least one housing disadvantage measure (overcrowding, eviction, mortgage delinquency, subjective perceptions of poor housing, housing mobility, tenure, or physical conditions) and mental health, including depression, and concluded that previous exposure to housing disadvantage may affect mental health later in life [75].

The presence of psychiatric/psychological conditions was also found to be linked with depression. They were discussed in a number of studies including low self-esteem and optimism, shame, neuroticism, negative self-concept, emotionality, and thinking, sensitivity to rejection, and others. Some of which was mediated by variables that include limited problem-solving ability, lack of social support, and rumination. However, determinants for depression may be present in several forms: as mediators or moderators, risk factors, or outcomes, and for this reason it is difficult to disentangle the relationships between the various factors related to depression [76].

Exposure to work and family stressors, as predictors for depression, cannot be overlooked. Marchand et al. investigated the mediating role of family-to-work conflict (FWC) and work-to-family conflict (WFC) and possible gender differences in exposure and vulnerability. They found WFC played a mediating role between work-family stressors and depression with strong association with women depressive symptoms. Irregular work schedule, higher working hours, and skill utilization acted as mediators [77]. In their systematic review, Dyrbye et al. found a high prevalence of depression among medical students, with levels of overall psychological distress consistently higher than in the general population [78].

It is worthy to note that the association between depression and both social media usage and smartphone addiction was also reported. Social media's link to mental health may be explained by the displaced behavior hypothesis. Long-term use of social networking platforms may be associated with depressive symptoms. Additionally, social media users may feel a lot of pressure to fit in with stereotypes and become as famous as others [79]. A bidirectional relationship between social media use and depression was also suggested [80].

In 2014, in Saudi culture, the demand for mental health treatment was still not seen as urgent. Even though the government controls a large portion of the health care system, organized psychiatric treatments are being delivered more quickly in the private sector. Despite much research showing the importance of early detection and cost savings of up to 80%, physicians in primary care continue to miss up to 50% of depressive patients in their clinics [49]. Implementing strategies to overcome depression is vital to improving patients' well-being and maintaining the provision of high-quality mental health care.

### 4.3. Strengths and Limitations of Study

The main strengths of this analysis were that the results were reported in accordance with the PRISMA statement, most of the included studies had large sample sizes, there was no significant publication bias, articles were reviewed, and data were extracted by independent investigators to obtain accurate results. Nevertheless, several limitations are yet to be mentioned. First, the analysis relied on aggregated published data. Important data might be missing from eligible articles that we could not search for or did not receive responses from their authors. Second, significant heterogeneity exists between studies with deficient data to define the sources of such heterogeneity. However, the variability in outcome estimates might be due to differences in population and study characteristics, cultural variations, the use of different depression scales and cut-off values that may affect the diagnostic sensitivity and specificity, associated risk factors or chronic conditions, the impact of COVID-19 in recent studies, sampling and data recruitment strategies, statistical methods, etc. Accordingly, the results should be interpreted with caution considering the potential impact on the validity of the findings. Further, we conducted a subgroup analysis to investigate sources of heterogeneity and adjusted analysis to take into account the variance due to different tools. However, some characteristics that may further explain heterogeneity were not reported or there were not enough data to conduct such analysis including age groups, cultural variations, working environments, and exposure to stress which may nonetheless have an effect on the prevalence of depression. Third, some administrative areas were not studied including the Northern Borders, Madinah, Al-Jawf, and Asir areas; hence, understanding the burden of depression in some areas within the country is restricted. Fourth, we were unable to unify the assessment criteria for prevalence with substantial study-level differences. Fifth, we excluded studies with a sample size of less than 100 participants which may have unintended consequences with potential bias in the results. We aimed to ensure an adequate level of statistical power and robustness in our analysis and reduce the risk of bias as including studies with small sample sizes can increase the risk of type II and type I errors, leading to false-negative or false-positive results and inaccurate conclusions. Additionally, studies with small sample sizes may have limited generalizability and may not meet the minimum quality criteria required for inclusion in a systematic review. To address these concerns, a sensitivity analysis was conducted to assess the impact of this exclusion criterion on the findings.The results suggested that the inclusion ofindividual studies with small sample sizes affected the overall prevalence estimate by more than 1%. Sixth, age is an important factor that may influence the prevalence of depression. However, there was no sufficient data to pool the prevalence of depression among different age groups. Seventh, the data reviewed were mostly self-reported via a cross-sectional design and involved screening rather than diagnostic depression tools that may limit accuracy for classifying patients with depression. Eighth, the prevalence, as a disease burden measure, may not be entirely accurate (point prevalence) and may change over time so, it needs to be studied from a longitudinal perspective. Finally, meta-analysis is not free of potential bias due to models, estimations, study selection, and publication bias. Future nationwide longitudinal research utilizing a single validated tool to measure depression in a random subset of individuals would provide a more precise estimate of the prevalence of depression among Saudi adults. Despite limitations, our analysis gives insight into some important aspects related to estimation of depression among Saudi adults that may have potential influences on providing psychological care, adopting preventive strategies, and directing decisions about who to prioritize for surveillance and intervention activities for depression management.

## 5. Conclusion

Our analysis showed that almost more than one third Saudi adults had depression. Female gender, being single, low education level, financial problems, poor housing condition, having medical problems, sleep disorders, presence of psychiatric/psychological conditions, life events, lack of social support, exposure to stress, educational/personal problems, and smartphone addiction were the independent predictors of depression among adults in Saudi Arabia. Appropriate surveillance, early interventions, and improving access to information may help to decrease the prevalence of depression, address disparities across different cohorts, develop cost-effective management strategies especially among adults, and prioritize depression in existing healthcare system.

## Figures and Tables

**Table 1:** Participants' characteristics of all studies included in the meta-analysis.

Study characteristics							Total population characteristics			Participants with depression							
Author (year of publication)	Study area (region)	Study setting	Target population	Depression tool	RR (%)	N-O checklist score	Sample size	Age mean (SD)/median (IQR)	Gender M/F	Prev. no. (%)	Degree of depression				Age (year)	Gender M/F	Marital status Mar/Sig/other
											Mild	Mod.	Mod. severe	Severe			
AlHadi et al. (2022) [12]	Nationwide (all)	Healthcare	HCWs	PHQ-9	13	4	1253	34.05 (8.3)	504/749	609(48.6)	367	134	66	42		210/399	
Alrasheedi and Rabbani (2022) [13]	Qassim (central)	Healthcare	HCWs	DASS-21	53.8	3	119		90/29	59(49.6)							
Bahri et al. (2022) [14]	Jazan (southern)	Community/public	General population	DASS-21	100+	6	472	23 (21–26)	246/226	235 (49.8)	45	86		104			
Aziz et al. (2022) [15]	Jeddah (western)	Healthcare	HCWs	PHQ-9	91.4	3	256	35.7 (6.9)	72/184	180 (70.3)	87	44	38	11			
Bondagji et al. (2022) [16]	Makkah (western)	Healthcare	HCWs	HADS	43	3	258	29	143/115	54(20.9)					24 − 26 = 12 27 − 31 = 20 32 − 36 = 16 >36 = 6	36/18	34/19/1
AlHamad and AlAmri (2021) [17]	Riyadh (central)	Community/public	General population	PHQ-9	100+	6	467	27 (10.9)	269/198	347 (74.3)	160	113	47	27			
Rizg et al. (2021) [18]	Jeddah (western)	University	University students	DASS-21	13.3	3	294		134/160	34 (11.6)						6/28	
Wali et al. (2021) [19]	Jeddah (western)	University	University students	PHQ-9	100	5	290		163/127	216 (74.5)	93	63	38	22		110/106	
Alharbi (2021) [20]	Riyadh (central)	University	University students	BDI	50	4	150		0/150	43 (28.7)					18 − 21 = 25 22 − 25 = 11 26 − 29 = 7	0/43	8/34/1
Anwar et al. (2021) [21]	Hail (northern)	University	University students	BDI	100	3	155	22.6 (1.3)	78/77	91 (58.7)	41	30	19	1		43/48	
Almoammar et al. (2021) [22]	Albaha (southern)	University	University students	PHQ-9	72.6	4	218	23.1 (1.54)	92/126	194 (88.99)	48	68	45	33			
Almarhapi and Khalil (2021) [23]	Tabuk (northern)	Healthcare	HCWs	PHQ-9	100+	3	177	30.6 (5.3)	60/117	71 (40.11)	47	20		4			
Mirza et al. (2021) [24]	Makkah (western)	University	University students	DASS-21	100+	6	465	21.46 (2.0)	198/267	251 (53.97)	60	98	48	45			
Alshardi and Faraha (2020) [25]	Jeddah (western)	Healthcare	HCWs	PHQ-9	72.8	3	149		79/70	113 (75.8)	53	51		9	≤26 = 55 >26 = 58	51/62	45/68/0
Alothman et al. (2021) [26]	Riyadh (central)	Healthcare	HCWs	CES-D	70	4	119		70/49	70 (58.8)	24		46		<30 = 60 >30 = 10	39/31	28/39/3
AlShamlan et al. (2021) [27]	Dammam (eastern)	University	University students	PHQ-9	96.5	6	527	22.4 (0.96)	239/288	362 (68.7)	155	112	64	31		140/222	45/310/7
Alzahrani et al. (2021) [28]	Riyadh (central)	University	University students	PHQ-9	85	3	289	21.5 (1.6)	140/149	208 (72.0)	96	59	27	26			
Mohammad et al. (2021) [29]	Makkah (western)	University	University students	DASS-21	100	5	373	22 (1.11)	373/0	186 (49.86)	85	62	17	22		186/0	
Alkhamees et al. (2021) [30]	Qassim (central)	University	University students	PHQ-9	87.1	3	305		144/161	153 (50.2)		73	39	41	18 − 21 = 52 >22 = 101	46/107	9/144/0
Desouky and Abu-Zaid (2021) [31]	Taif (western)	University	University students	BDI	74.5	6	1513	20.58 (1.71)	688/825	494 (32.65)	220	249		25		206/288	
Al Balawi et al. (2019) [32]	Tabuk (northern)	Community/Public	General population	PHQ-9	100	5	384		90/294	284 (74.0)	145	80	29	30	20 − 25 = 132 26 − 30 = 49 31 − 35 = 41 36 − 40 = 62	60/224	107/166/11
Hamasha et al. (2019) [33]	Jeddah (western)	University	University students	BDI	56.9	3	398		208/190	178 (44.7)	82	70		26			
AlFaris et al. (2019) [34]	Riyadh (central)	University	University students	BDI	81	6	460	21.04 (1.55)	234/226	209 (45.4)	108	73		28		101/108	
AlFaris et al. (2019) [35]	Riyadh (central)	Healthcare	HCWs	BDI	76	3	186	26.9	87/99	56 (30.1)	15	26		15		17/39	
Albajjar and Bakarman (2019) [36]	Albaha (southern)	University	University students	BDI	100	4	182	22.03 (1.94)	182/0	98 (53.8)	62	28		8		98/0	15/83/0
Al Rashed et al. (2019) [37]	Al-Ahsa (eastern)	Community/Public	General population	PHQ-9	96.3	6	5172		2124/3048	447 (8.6)						152/295	
Almalki et al. (2019) [38]	Riyadh (central)	University	University students	BDI	51.3	3	241	21.7 (2.6)	152/89	142 (58.92)	46	28	38	30		74/68	
Bahhawi et al. (2018) [39]	Jazan (southern)	University	University students	DASS-21	90.16	6	642	22.14 (1.7)	328/314	439 (68.38)	95	214	67	63			
AlFahhad (2018) [40]	Riyadh (central)	Healthcare	HCWs	PHQ-9	100	4	300	33 (8.6)	201/99	66 (22.0)							
Alsharif et al. (2018) [41]	Jeddah (western)	Healthcare	HCWs	BDI	60.9	4	443	37	0/443	55 (12.4)	42	11		2	20 − 29 = 12 30 − 39 = 21 40 − 49 = 20 50 − 59 = 2	0/55	47/4/4
Alhassan et al. (2018) [42]	Nationwide (all)	Community/Public	General population	BDI	83.5	6	935	31.7 (10.98)	316/619	327 (35.0)	112	140		75			
Dawood et al. (2017) [43]	Riyadh (central)	University	University students	BDI	79.7	3	149	21.57 (1.16)	0/149	52 (34.89)	27	4	12	9			
Alsalameh et al. (2017) [44]	Nationwide (all)	University	University students	PHQ-9	100+	6	1171		433/738	723 (61.74)	301	211		211		241/482	
Basudan et al. (2017) [45]	Riyadh (central)	University	University students	DASS-21	95.8	3	247		134/113	138 (55.87)	35	53	21	29			
AlFaris et al. (2016) [46]	Riyadh (central)	University	University students	BDI	79	6	1186	21.34 (1.58)	668/518	259 (21.84)	122	91		46		104/155	
Jarwan (2015) [47]	Makkah (wwwestern)	University	University students	BDI	100	3	136	20.9 (1.1)	63/73	42 (30.9)	25	13		4		17/25	
Al Zahrany et al. (2015) [48]	Taif (western)	Community/Public	General population	BDI	100	4	165	47.5 (13.9)	76/89	71 (43.0)	36	27		8	<30 = 7 30 − 49 = 33 50 − 59 = 31	19/52	56/9/6
Al-Qadhi et al. (2014) [49]	Riyadh (central)	Community/Public	General population	PHQ-9	86.7	3	477	38 (12)	161/316	238 (49.9)	148	64	21	5			
Joury et al. (2014) [50]	Riyadh (central)	Community/Public	General population	BDI	75.4	3	527		241/286	175 (33.2)	71	79		25			
Amr et al. (2013) [51]	Al-Ahsa (eastern)	University	University students	PHQ-9	100+	7	1696	20.8 (1.9)	1072/624	910 (53.7)	413	329		168		514/396	
Al-Amri and Al-Amri (2013) [52]	Taif (western)	King Fahad Air Base	Military personnel	BDI	100	4	357	33.1 (7.4)	357/0	61 (17.1)	45	12		4			48/13/0
Ibrahim et al. (2013) [53]	Jeddah (western)	University	University students	HADS	100+	4	426	21.1 (1.4)	0/426	58 (13.6)							
AL-Faris et al. (2012) [54]	Riyadh (central)	University	University students	BDI	95	6	796	21.63 (1.57)	590/206	384 (48.2)	165	132		87		265/119	13/367/4
Abbas et al. (2012) [55]	Riyadh (central)	Healthcare	HCWs	HADS	55	3	715	35.2 (8.2)	83/632	69 (9.65)					20 − 29 = 21 30 − 39 = 35 40 − 49 = 13	22/47	44/18/7
Abdelwahid and Al-shahrani (2011) [56]	Sharurah (southern)	Community/Public	General population	PHQ-9	97	4	272	29.9 (7.1)	116/156	33 (12.1)	25	3	3	2		24/9	27/6/0
Inam (2007) [57]	Qassim (central)	University	University students	AKUADS	83.9	3	302		198/104	151 (50.0)						88/63	

AKUADS: The Aga Khan University Anxiety and Depression Scale; BDI: Beck Depression Inventory; CES-D: The Center for Epidemiological Studies-Depression; DASS-21: Depression Anxiety and Stress Scale; F: female; HADS: Hospital Anxiety and Depression Scale; HCWs: healthcare workers; IQR: interquartile range; M: male; Mar: married; Mod.: moderate; N-O: Newcastle-Ottawa scale; PHQ-9: Patient Health Questionnaire; Prev.: prevalence; RR: response rate; SD: standard deviation; Sin: single.

**Table 2:** Associated factors and predictors for depression among Saudi adults.

Author (year of publication)	Associated factors	Strength of association
AlHadi et al. (2022) [12]	Female gender	OR = 1.81, 95% CI (1.33–2.45), *p* > 0.001

Alrasheedi and Rabbani (2022) [13]	Not passing the promotion exam in the first attempt	AOR = 4.43, 95% CI (1.45–13.5)
	Internal medicine speciality compared to family medicine	AOR = 3.94, 95% CI (1.22–12.66)

Bahri et al. (2022) [14]	Female gender	OR = 1.8, 95% CI (1.2–2.7), *p* = 0.002

Aziz et al. (2022) [15]	Female gender	OR = 4.66, 95% CI (1.56–13.87), *p* = 0.006
	Presence of work stressor	OR = 3.08, 95% CI (1.15–8.24), *p* = 0.025

Bondagji et al. (2022) [16]	Far from enough sleep amount	OR = 3.3, 95% CI (1.0–11.2), *p* = 0.050
	Injustice in workplace	OR = 2.0, 95% CI (1.2–3.3), *p* = 0.010
	Loneliness	OR = 2.7, 95% CI (1.7–4.4), *p* < 0.001

AlHamad and AlAmri (2021) [17]	Female gender	OR = 1.48, *p* = 0.040
	Basic social media usage	r =0.211, *p* < 0.001
	Social media interaction usage	r =0.100, *p* = 0.031
	Social media display usage	r =0.161, *p* < 0.001

Almarhapi and Khalil (2021) [23]	Loosing beloved person in the last 6 months	AOR = 3.67, 95% CI (1.84–7.3), *p* < 0.001

Mirza et al. (2021) [24]	Female gender	AOR = 2.13, 95% CI (1.15–3.93), *p* = 0.020
	Presence of family conflicts	AOR = 1.83, 95% CI (1.02–3.26), *p* = 0.040
	Presence of psychiatric condition	AOR = 10.66, 95% CI (1.32–86.38), *p* = 0.030
	Travel time from home to university	AOR = 1.5, 95% CI (1.02–2.19), *p* = 0.040
	Being at senior academic year	AOR = 2.53, 95% CI (1.29–4.95), *p* = 0.010

Alshardi and Faraha (2020) [25]	Female gender	AOR = 2.12, 95% CI (0.93–4.81), *p* = 0.070
	Being single	AOR = 2.81, 95% CI (1.13–7.01), *p* = 0.030
	Having medical problem	AOR = 9.45, 95% CI (1.12–80.05), *p* = 0.040
	Being at a higher residency level	AOR = 6.14, 95% CI (1.31–28.93), *p* = 0.020
	Residents in surgery program	AOR = 2.6, 95% CI (1.13–5.98), *p* = 0.030
	Residents in emergency program	AOR = 4.9, 95% CI (1.08–22.2), *p* = 0.040

AlShamlan et al. (2020) [27]	Being not ready for speciality	OR = 2.24, 95% CI (1.21–4.1), *p* = 0.009
	Female gender	OR = 1.91, 95% CI (1.24–2.93), *p* = 0.003

Alzahrani et al. (2020) [28]	Exposure to stress	OR = 1.26, 95% CI (1.17–1.36), *p* < 0.001

Alkhamees et al. (2020) [30]	Female gender	AOR = 4.77, 95% CI (2.88–7.91), *p* < 0.05
	Exposure to high emotional exhaustion	AOR = 6.54, 95% CI (3.59–11.9), *p* < 0.05
	Exposure to high cynicism	AOR = 4.98, 95% CI (2.21–11.22), *p* < 0.05

Al Balawi et al. (2019) [32]	Lack of social support	AOR = 2.05, 95% CI (1.03–4.07), *p* = 0.041
	Disturbed marriage	AOR = 3.5, 95% CI (1.23–9.98), *p* = 0.019
	Financial problems	AOR = 2.37, 95% CI (1.16–4.85), *p* = 0.019
	Stressful experience	AOR = 4.75, 95% CI (2.58–8.71), *p* < 0.001
	Family history of depression	AOR = 2.75, 95% CI (1.23–6.14), *p* = 0.014
	Sleep disorders	AOR = 2.24, 95% CI (1.16–4.3), *p* = 0.016

Hamasha et al. (2019) [33]	Presence of psychological problem	OR = 4.2, 95% CI (1.15–15.34), *p* = 0.030
	Poor social life	OR = 2.36, 95% CI (1.62–3.44), *p* < 0.001
	Recent loss of family members	OR = 2.12, 95% CI (1.11–4.07), *p* = 0.024

Al Rashed et al. (2019) [37]	Having difficulties at work and home	AOR = 5.8, 95% CI (4.2–8.05), *p* < 0.001
	Lower education level	AOR = 1.5, 95% CI (1.21–1.88), *p* < 0.001

Alhassan et al. (2018) [42]	Low education level	*β* = −2.034, adjusted *p* = 0.010
	Higher smartphone addiction	*β* = 0.194, adjusted *p* < 0.001

Dawood et al. (2017) [43]	Low GPA scores	*r* = −0.224, *p* = 0.006
	Less educated parents	*r* = −0.203, *p* = 0.013
	Low satisfaction with social support	*r* = −0.520, *p* < 0.001

Basudan et al. (2017) [45]	Low satisfaction with relationship with peers	*β* = −0.229, *p* < 0.001
	Low satisfaction with relationship with college faculty	*β* = −0.174, *p* = 0.007

Jarwan (2015) [47]	History of loss of first-grade relative in the last year	OR = 3.23, 95% CI (1.08–9.68)
	History of depression	OR = 3.53, 95% CI (1.46–8.51)

Amr et al. (2013) [51]	Nature of the educational stream	OR = 1.01, 95% CI (1.01–1.4), *p* < 0.05
	Female gender	OR = 2.12, 95% CI (1.3–3.44), *p* < 0.001
	Presence of financial problems	OR = 1.73, 95% CI (1.00–3.06), *p* < 0.05
	Presence of educational problems	OR = 2.26, 95% CI (1.42–3.61), *p* < 0.05
	Presence of personal problems	OR = 2.12, 95% CI (1.29–3.48), *p* < 0.05

Al-Amri and Al-Amri, 2013 [52]	Having female siblings only	OR = 2.74, 95% CI (1.05–7.17), *p* = 0.040
	Having problems with relation with supervisors	OR = 6.17, 95% CI (1.13–33.95), *p* = 0.040
	Having problems with relation with relatives	OR = 3.58, 95% CI (1.47–8.79), *p* = 0.005

Ibrahim et al. (2013) [53]	Condensed courses	OR = 1.9, 95% CI (1.04–3.45), *p* = 0.030
	Feeling no cooperation	OR = 2.14, 95% CI (1.24–3.67), *p* = 0.005
	Emotional failure	OR = 1.78, 95% CI (1.04–3.15), *p* = 0.030
	History of loss of a close friend	OR = 2.0, 95% CI (1.11–3.64), *p* = 0.010
	Presence of anxiety	OR = 3.28, 95% CI (1.85–5.82), *p* < 0.001

Abdelwahid and Al-Shahrani (2011) [56]	Occupational status (employee vs. nonemployee)	OR = 1.65, 95% CI (1.14–20.30), *p* = 0.030
	Housing condition (living in a room vs. flat or villa)	OR = 4.82, 95% CI (1.28–18.09), *p* = 0.020

Inam (2007) [57]	Female gender	OR = 5.8, 95% CI (1.3–28.7), *p* = 0.006

AOR: adjusted odds ratio; *β*: beta standardized coefficient; GPA: grade point average; OR: odds ratio; *r*: correlation coefficient.

## Data Availability

The datasets used and analyzed for the current study are available from the corresponding author upon reasonable request. The confidentiality and security of data and materials were ensured through all stages of the study.

## Abbreviations

AKUADS: The Aga Khan University Anxiety and Depression Scale
AOR: Adjusted odds ratio
BDI: Beck Depression Inventory
CES-D: The Center for Epidemiological Studies-Depression
CI: Confidence interval
DALYs: Disability-adjusted life-years
DASS-21: Depression Anxiety and Stress Scale-21
FWC: Family-to-work conflict
GBD: Global Burden of Diseases Study
HADS: Hospital Anxiety and Depression Scale
IQR: Interquartile range
MOH: Ministry of Health
OR: Odds ratio
PHQ-9: Patient Health Questionnaire
PRISMA: Preferred Reporting Items for Systematic Reviews and Meta-Analyses
PROSPERO: International prospective register of systematic reviews
SD: Standard deviation
SDL: Saudi Digital Library
WFC: Work-to-family conflict
WHO: World Health Organization.
